# Efficacy of Dupilumab in Patients with Chronic Rhinosinusitis with Nasal Polyps and Eosinophilic Otitis Media: A Six-Month Observational Study

**DOI:** 10.3390/medicina61081471

**Published:** 2025-08-15

**Authors:** Cosimo Galletti, Federica Giammona Indaco, Daniele Portelli, Giulia Laterra, Patrizia Zambito, Maria Grazia Ferrisi, Leonard Freni, Francesco Ciodaro, Francesco Freni, Salvatore Maira, Bruno Galletti

**Affiliations:** 1 Otorhinolaryngology Unit, “Ospedale Umberto I” Enna, Faculty of Medicine and Surgery, Università degli Studi di Enna “Kore”, Contrada Ferrante, 94100 Enna, Italy; samedico@hotmail.it; 2 Otorhinolaryngology Unit, Ospedale “Umberto I” Enna, Contrada Ferrante, 94100 Enna, Italy; federicagiammonaindaco@gmail.com (F.G.I.); ,; 3 Unit of Otorhinolaryngology, Department of Adult and Development Age Human Pathology “Gaetano Barresi”, University of Messina, Via Consolare Valeria 1, 98125 Messina, Italy; daniele.portelli09@gmail.com (D.P.); mariagraziaferrisi58@gmail.com (M.G.F.); leonardfreni@gmail.com (L.F.); dottfciodaro@alice.it (F.C.); franco.freni@tiscali.it (F.F.); bgalletti@unime.it (B.G.); 4 Cardiology Unit Umberto I Enna, Faculty of Medicine and Surgery, Università degli Studi di Enna “Kore”, Contrada Ferrante, 94100 Enna, Italy; giulia.laterra@unikore.it (G.L.)

**Keywords:** CRSwNP, EOM, dupilumab, rhinology, otology

## Abstract

*Background and Objectives:* Chronic rhinosinusitis with nasal polyps (CRSwNP) and eosinophilic otitis media (EOM) are frequently co-existing eosinophilic disorders related to type 2 inflammation, which significantly impair the quality of life of patients. Dupilumab, a monoclonal antibody targeting the IL-4 receptor alpha and anti-IL-13, has demonstrated a promising profile of efficacy and safety in the treatment of CRSwNP; however, evidence on its role in concomitant EOM and CRSwNP remains limited in the literature. This study aims to evaluate the clinical efficacy of dupilumab in patients with concomitant CRSwNP and EOM over a six-month observational period. *Materials and Methods:* A retrospective observational cohort study was conducted on twenty-two patients (aged 18–75 years) over six months with severe uncontrolled CRSwNP and confirmed refractory EOM who were treated with dupilumab (300 mg every two weeks). Demographic data are collected, and outcome measures included Nasal Polyp Score (NPS), Sino-Nasal Outcome Test (SNOT-22), Visual Analog Scale for nasal congestion (VAS), tympanogram classification, and Chronic Otitis Media Outcome Test (COMOT-15), evaluated at baseline and 6 months. *Results*: Over the six-month treatment period, patients with coexisting CRSwNP and eosinophilic otitis media experienced significant improvements across the multiple validated clinical and patient-reported outcome measures. The Nasal Polyp Score (NPS) significantly decreased from a median of 5.7 (IQR: 1.2) at baseline to 1.5 (IQR: 1.3) at six months (*p* < 0.0001). The SNOT-22 showed a substantial decline from a median of 77.6 (IQR: 19.0) to 21.5 (IQR: 13.4), *p* < 0.0001. Visual Analog Scale (VAS) scores for nasal congestion improved significantly from 8.4 (IQR: 1.1) to 1.7 (IQR: 1.2), *p* < 0.0001. Tympanogram scores improved from Tympanogram type “B” to Tympanogram type “A” (*p* = 0.018). COMOT-15 scale decreased from a median of 51.3 (IQR: 8.4) to 19.2 (IQR: 5.0) (*p* < 0.0001). Peripheral eosinophil counts remained unchanged or increased (baseline 0.80 vs. 0.84 cells/μL at six months, (*p* = 0.834)). *Conclusions*: Dupilumab treatment in patients with CRSwNP and EOM led to significant clinical improvements in sinonasal symptoms, middle ear function, and quality of life over six months, with no significant change in peripheral eosinophilia.

## 1. Introduction

Chronic rhinosinusitis with nasal polyps (CRSwNP) and eosinophilic otitis media (EOM) are both manifestations of type 2 (Th2-mediated) inflammation, characterized by elevated levels of interleukin (IL)-4, IL-5, and IL-13, leading to eosinophilic infiltration of mucosal tissues [1,2]. CRSwNP affects 2–4% of the adult population and often coexists with asthma and allergic rhinitis, forming a unified airway inflammatory disease [3,4]. Similarly, EOM is a recurrent and severe form of otitis media characterized by a viscous, eosinophil-rich effusion, commonly associated with asthma and nasal polyposis. It is often resistant to conventional treatments, including tympanostomy tubes and topical or systemic corticosteroids [5,6,7], and it refers to inflammation or infection of the middle ear, which can be acute or chronic. Chronic otitis media often occurs with upper respiratory conditions, including chronic rhinosinusitis with nasal polyps (CRSwNP), because the sinuses and the ear are connected through the Eustachian tube in a common morphofunctional unit. Eosinophils play an important role in the EOM. Like in CRSwNP and Severe Eosinophilic asthma (SEA), eosinophils migrate to inflammatory sites, activate various cytokines and chemokines, and release cytotoxic granules contributing to tissue damage, cell death, and chronic inflammation with remodeling effects on the nasal mucosa. Patients with both CRSwNP and OM experience important morbidity symptoms, including ear pain, hearing loss, and ear fullness.

EOM represents a high risk for the development of a serious otological disease, and the concomitant CRSwNP worsens the quality of life (QoL) of these patients.

According to the literature [2], EOM is classified as follows: major criteria like otitis media with effusion or chronic otitis media with eosinophil-dominant effusion, and two or more minor criteria, like highly viscous middle ear effusion and resistance to conventional treatment for otitis media.

Dupilumab, a fully human monoclonal antibody targeting the IL-4 receptor alpha subunit, inhibits IL-4 and IL-13 signaling, thereby reducing the downstream effects of type 2 inflammation (Th2) [8]. Clinical trials such as SINUS-24 and SINUS-52 have demonstrated that dupilumab significantly reduces nasal polyp burden, improves nasal airflow, and enhances health-related quality of life in patients with severe CRSwNP [9,10]. However, its efficacy in EOM remains largely underexplored, despite the shared immunopathologic mechanisms with CRSwNP.

Recent evidence suggests that targeted biologic therapy may offer a new treatment paradigm in managing complex, multi-compartment eosinophilic disease, particularly in patients with overlapping upper and middle airway involvement [11]. Given the chronic and relapsing nature of both CRSwNP and EOM, along with their substantial impact on hearing, olfaction, and overall quality of life, an integrated approach to evaluation and management is essential [12].

This study investigates the clinical efficacy of dupilumab over six months in patients with coexisting CRSwNP and EOM, using objective and subjective outcome measures including the Nasal Polyp Score (NPS), SNOT-22, tympanograms, and the Chronic Otitis Media Outcome Test (COMOT-15), a validated tool for assessing health-related quality of life in otologic disorders [13].

This study aims to report the results obtained with therapy in patients with CRSwNP enrolled and followed up in two centers in Sicily. The primary aim of this study was to evaluate the effectiveness of dupilumab during the first six months of treatment in a real-life setting, focusing on improvement in CRSwNP and EOM.

## 2. Materials and Methods

We collected data from patients affected by CRSwNP and EOM who were followed by “Umberto I” Hospital, ENT Department, Kore University of Enna reference Hospital, and “G.Martino” University Hospital of Messina, ENT Department, University of Messina, performing a multicentric observational cohort study, to assess the effectiveness and safety of dupilumab. The study population was collected from January 2024 to April 2025. The population included patients, male or female, ≥18 years with a diagnosis of severe refractory CRSwNP who had received systemic and/or topical corticosteroids in the preceding two years, previous sinonasal surgery, or not, and a confirmed diagnosis of EOM. We follow the diagnostic criteria for severe CRSwNP established from the European Position Paper on Rhinosinusitis and Nasal Polyps (EPOS 2020) and EUFOREA. We defined refractory eosinophilic otitis media according to the diagnostic criteria established by Iino et al. [2]. The study sample included patients’ willingness and ability to provide written informed consent, with an observational follow-up period of at least 6 months

We excluded patients who had low adherence to the therapeutic protocol, underwent radio-chemotherapy treatment in the last 12 months, or were affected by chronic autoimmune disease treated with long-term systemic corticosteroid, as well as patients who reported a tympanic membrane perforation, and females who were pregnant.

We preserve the anonymity of patients using an encrypted code in agreement with the Declaration of Helsinki.

We performed a retrospective analysis of data at the baseline and the 6-month follow-up visit from the start of monoclonal therapy. We collected demographics data as sex, smoking habits, topic and systemic corticosteroid intake, previous endoscopic sinus surgery and the number, comorbidity as asthma, IgE serum level, allergy to nonsteroidal anti-inflammatory drugs (NSAIDs), aspirin exacerbated respiratory disease (AERD), severity of CRSwNP, EOM criteria, and blood eosinophils.

We established the severity of disease by calculating the SNOT-22 questionnaire and performing an endoscopic sinonasal evaluation to assess the NPS. Blood IgE and eosinophils assessment was performed at the baseline and six months.

The therapeutic prescription of dupilumab following the criteria established by the Italian Medicines Agency (AIFA) for patients affected by CRSwNP. The date of the first injection is considered the “index date”. Every 14 days the patient was subjected to an injection of dupilumab 300 mg before a regulated follow-up visit at six-months to evaluate the outcome measures:

- SNOT-22: We used the validated Italian version of SNOT-22. Possible total score range: 0–110. A SNOT-22 score < 20 was suggestive of mild symptoms. During follow-up time, the minimal clinically important difference (MCID) in SNOT-22 scores was assumed for an 8.9-point increase as reported in previous studies.
- Nasal endoscopy (Nasal Polyp Score): Each side of the nasal cavity was separately evaluated and scored in a range from 0 to 4 (0 = no polyps; 1 = small polyps in the middle meatus not reaching below the inferior border of the middle turbinate; 2 = polyps reaching below the lower border of the middle turbinate; 3 = large polyps reaching the lower border of the inferior turbinate or polyps medial to the middle turbinate; and 4 = large polyps causing complete obstruction of the inferior nasal cavity). The sum of the scores for both nasal cavities was recorded as the NPS value.
- VAS for symptoms: Intensity of symptoms was measured on a horizontal 10 cm line. A mean score for each symptom analyzed was obtained using the average scores assigned to all patients for the same symptom.
- Diagnostic Criteria of Eosinophilic Otitis Media (EOM): Major criteria: Otitis media with effusion or chronic otitis media with eosinophilic dominant effusion. Minor criteria: Highly viscous middle ear effusion, resistance to conventional treatment for OM, association with asthma, and association with CRSwNP. Definitive case: Positive for major criteria + two or more minor criteria. Exclusion criteria: Eosinophilic Granulomatosis with Polyangiitis (EGPA) [2].
- Tympanogram: “A”, “B” or “C”.
- Chronic otitis media outcome test (COMOT-15): We used the validated Italian version of COMOT-15. The COMOT-15 consists of three subscales, categorized as ear symptoms (questions 1–6), hearing function (questions 7–9), and mental health (questions 10–13), which form the overall score. In addition to questions 1 to 13, the COMOT-15 contains two other questions: a general evaluation of the impact of chronic otitis media on QoL (question 14) and a question on the frequency of ENT visits as a result of chronic otitis media in the previous 6 months (question 15). The possible total score range is 0–75 [COMOT] [13].

We performed a descriptive data analysis with StatPlus: mac V8 version. We calculated the medians with interquartile ranges (Pre-Post) for continuous variables; continuous variables were estimated with absolute and percentage frequencies. All endoscopic evaluations by NPS and subjective perception comparisons by SNOT-22 were made between data obtained at the baseline and the 6th month. Statistical significance was assumed for *p* values < 0.05.

## 3. Results

A total of 22 patients were included in the baseline cohort. The mean age was 55 years (±11.9), and 50% of the sample were male. Smoking was reported by 31.8% of participants. All patients (100%) were on chronic corticosteroid therapy, including oral corticosteroids (OCS), and had previously undergone endoscopic sinus surgery (ESS).

Comorbid asthma was present in 72.7% of the sample, while 90.9% reported allergic sensitization. Aspirin-exacerbated respiratory disease (AERD) and ASA triad were each identified in 40.9% of patients. The mean total serum IgE concentration at baseline was 108 IU/mL (±88.7) (Table 1).

Over the six-month treatment period, patients with coexisting CRSwNP and eosinophilic otitis media experienced significant improvements across multiple validated clinical and patient-reported outcome measures (Table 2).

**Figure 1:**
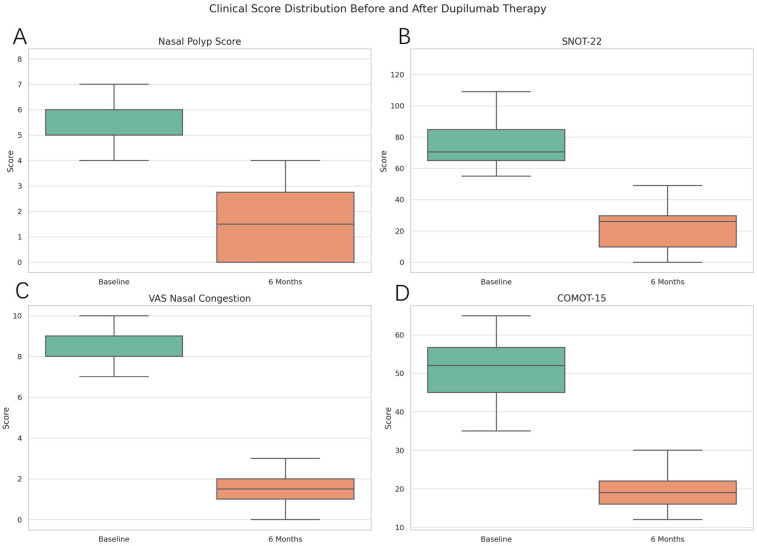
Changes in clinical parameters after 6 months of dupilumab therapy. Legend: the median and interquartile range of the scores. The plots show the distribution of individual patient data points. Subfigures (**A**–**D**) correspond to NPS, SNOT-22, VAS nasal congestion, and COMOT-15, respectively.

The Nasal Polyp Score (NPS) significantly decreased from a median of 5.7 (IQR: 1.2) at baseline to 1.5 (IQR: 1.3) at six months (*p* < 0.0001), indicating a marked reduction in endoscopic evidence of polyp burden. This decrease is visually represented in Figure 1, where post-treatment scores display a tighter distribution around lower values.

The SNOT-22, a validated tool for assessing sinonasal-related quality of life, showed a substantial decline from a median of 77.6 (IQR: 19.0) to 21.5 (IQR: 13.4), *p* < 0.0001. The corresponding boxplot (Figure 1) highlights a shift towards improved symptom burden, especially in domains related to nasal obstruction, facial pain, and sleep disturbance.

Visual Analog Scale (VAS) scores for nasal congestion improved significantly from 8.4 (IQR: 1.1) to 1.7 (IQR: 1.2), *p* < 0.0001, consistent with reduced obstruction and improved nasal airflow perception. Figure 1 illustrates this decline, underscoring the effectiveness of dupilumab in alleviating upper airway symptoms.

Improvements extended to otologic health. Tympanogram scores, indicating middle ear function, improved from a median of 2 (Tympanogram type “B”) (IQR: 1–2) to 1 (Tympanogram type “A”) (IQR: 0–1), *p* = 0.018. This shift suggests reduced effusion or better Eustachian tube function.

COMOT-15, a specific quality of life metric for otitis media, showed significant improvement, decreasing from a median of 51.3 (IQR: 8.4) to 19.2 (IQR: 5.0), *p* < 0.0001. Figure 1 reflects this change, which implies better hearing, less discomfort, and reduced impact on daily activities.

Peripheral eosinophil counts remained unchanged or increased (baseline 0.80 vs. 0.84 cells/μL at six months, *p* = 0.834), supporting the notion that the eosinophil plasmatic curve is strictly dependent on the dupilumab’s mechanism of action, which binds specifically to the IL-4Rα receptor subunit and thus blocks both IL-4 and IL-13 signaling. This transiently increases blood eosinophil concentrations by inhibiting eotaxin-3, resulting in a lack of migration of eosinophils from peripheral blood to polyp tissue, due to local anti-inflammatory activity rather than systemic eosinophil depletion. This finding may reflect localized rather than systemic inflammatory changes, or the possibility that eosinophil counts are not a sensitive marker for the observed clinical improvements in this cohort.

These results demonstrate dupilumab’s efficacy in simultaneously improving sinonasal and middle ear pathology, with broad implications for patient function and well-being.

## 4. Discussion

Eosinophilic otitis media (EOM) is a form of chronic otitis media characterized by the presence of eosinophils in the middle ear. EOM is recognized as a subtype of chronic otitis media with effusion (OME), and it is often associated with an allergic or inflammatory response pattern. The pathophysiology is thought to involve a hypersensitivity reaction leading to the recruitment of eosinophils into the middle ear, contributing to chronic inflammation and fluid accumulation [14].

On histologic examination, EOM shows a characteristic infiltration of eosinophils in the middle ear mucosa, often alongside other inflammatory cells, such as neutrophils and lymphocytes. This distinguishes EOM from other forms of chronic otitis media [14].

As we know, middle ear ventilation is strictly related to the status of the Eustachian tube, which serves as a conduit between the nasopharynx and tympanic cavity. Status of chronic inflammation in the nasal sinuses and nasal fossa could lead to chronic inflammation of the middle ear, particularly in patients with CRSwNP, where mechanical obstruction of the nasal fossa and the immunophlogosis of the sinonasal mucosa altered the nasal-cycle ventilation, affecting also the ventilation of the tympanic cavity [1]. This connection could explain why patients with CRSwNP are at a higher risk of developing middle ear pathology, including EOM. Our sample data and results suggest that there is a direct correlation between the severity of CRSwNP and EOM presentation [2,3].

Cytokines such as IL-5, eotaxin, IL4, IL-13, and TGF-β play a significant role in eosinophil recruitment and activation. Elevated levels of these cytokines are frequently observed in EOM patients and CRSwNP. Both diseases are believed to involve T-helper 2 (Th2) dominance, where the immune system’s overreaction to environmental allergens leads to the release of inflammatory mediators and recruitment of eosinophils. This Th2-driven immune response is a common link between CRSwNP and EOM [2,3].

Patients with both CRSwNP and EOM typically experience symptoms such as nasal congestion, rhinorrhea, post-nasal drip, ear fullness, hearing loss, tinnitus, and persistent effusive otitis media.

Standard therapeutic strategies mainly focus on treating nasal symptoms. In patients experiencing recurrent episodes of OME, myringotomy and/or the placement of tympanostomy tubes (grommets) are commonly used to drain the middle ear and prevent the progression of tympanic membrane degeneration and superinfection.

The development of targeted therapies with monoclonal treatment for CRSwNP significantly improves patients’ quality of life, highlighting the importance of regulating the Th2 inflammatory pattern in managing both diseases.

Our findings confirm and extend the current evidence based on dupilumab’s effectiveness in treating eosinophilic airway diseases, particularly in patients suffering from both CRSwNP and eosinophilic otitis media (EOM) [4,5,6]. The significant reductions observed in Nasal Polyp Score (NPS), SNOT-22, and nasal congestion VAS mirror those seen in pivotal clinical trials such as SINUS-24 and SINUS-52, where dupilumab was shown to reduce polyp size and improve health-related quality of life in patients with severe CRSwNP [4,15]. The fact that peripheral blood eosinophil levels remained unchanged during treatment supports the evidence that dupilumab primarily exerts its effects via local inhibition of IL-4 and IL-13 signaling within mucosal tissues. The remodeling effects on the nasal and sinonasal mucosa are correlated with dupilumab’s disease-modifying effect [7,14,15,16,17,18,19].

Importantly, our study adds new evidence suggesting that dupilumab may also have a beneficial effect on comorbid middle ear pathology. This is particularly relevant in EOM, a condition characterized by persistent middle ear effusion, conductive hearing loss, and a high recurrence rate due to eosinophilic inflammation [2,18]. The observed improvements in tympanogram scores indicate potential enhancement of Eustachian tube function or reduction in middle ear inflammation, both of which are clinically significant.

COMOT-15 is especially valuable as a patient-reported outcome measure, reflecting physical discomfort, functional limitations, and emotional distress associated with otitis media. Furthermore, the significant decrease in COMOT-15 scores highlights dupilumab’s important impact on auditory symptoms and related quality of life issues [8,9]. The COMOT-15, developed by Baumann et al. [8] is a validated instrument that captures physical symptoms (e.g., hearing loss, ear discharge), functional limitations, and emotional impact. Its sensitivity to treatment-induced changes makes it a valuable tool for investigating therapeutic response in otologic disease. In a study [5], COMOT-15 scores were shown to correlate with both objective otoscopic findings and subjective symptom burden in EOM patients, reinforcing its utility.

Moreover, dupilumab demonstrates a therapeutic effect in patients with “united airway disease”, given the shared type 2 inflammatory endotype of CRSwNP, asthma, and EOM [20,21,22]. Targeting a common cytokine pathway may simultaneously alleviate symptoms across multiple anatomical sites, improving overall disease control and reducing the need for systemic corticosteroids and surgery [11].

Despite these promising results, limitations of the current study include its observational design, lack of a control group, and relatively small sample size. Furthermore, we do not assess the olfactory function outcome measure and include at least a subjective olfactory outcome measure with the Snot-22 questionnaire. Also, we do not concentrate our attention on improvement in the asthma condition due to the focus of our study: otitis media. Another limitation of the current study could be the population selection, because our patients were affected by both CRSwNP and EOM, resulting in a more expressive Th2 pattern of inflammation, subsequently, with better results for the treatment with dupilumab.

Future studies are warranted to further elucidate the long-term otologic benefits of dupilumab, ideally in larger, prospective cohorts. In particular, incorporating objective audiologic assessments and imaging of the middle ear is needed to fully characterize dupilumab’s otologic benefits.

## 5. Conclusions

Dupilumab treatment in patients with CRSwNP and EOM led to significant clinical improvements in sinonasal symptoms, middle ear function, and quality of life over six months, with no significant change in peripheral eosinophilia. These results support dupilumab as a promising therapeutic option in patients with CRSwNP and Eosinophilic Otitis Media.

## Figures and Tables

**Table 1:** Population Sample General Characteristics.

	Population Sample at the Baseline (*n* = 22)
**Mean Age, y**	55 ± 11.9
**Male**	11 (50%)
**Smoking**	22 (31.8%)
**CCS**	22 (100%)
**OCS**	22 (100%)
**Previous ESS**	22 (100%)
**Asthma**	16 (72.7%)
**Allergy**	20 (90.9%)
**AERD**	9 (40.9%)
**ASA TRIAD**	9 (40.9%)
**Mean IgE**	108.8 ± 88.7

**Table 2:** Changes in clinical outcome measures from baseline to 6 months.

Outcome Measure	Baseline Median (IQR)	6-Months Median (IQR)	*p*-Value (95% CI)	Test Used
**Mean EOS (0.1 × 10^3^/μL)**	0.80 ± 0.49	0.84 ± 0.58	0.834 (−0.38–0.31)	Wilcoxon signed-rank
**Mean NPS**	5.7 ± 1.2	1.5 ± 1.3	<0.0001 (3.61–4.76)	Wilcoxon signed-rank
**Mean SNOT 22**	77.6 ± 19.0	21.5 ± 13.4	<0.0001 (45.13–66.96)	Wilcoxon signed-rank
**Mean VAS**	8.4 ± 1.1	1.7 ± 1.2	<0.0001 (5.92–7.54)	Wilcoxon signed-rank
**Mean COMOT 15**	51.3 ± 8.4	19.2 ± 5.0	<0.0001 (28.90–35.28)	Wilcoxon signed-rank
**Mean EOS**	0.80 ± 0.49	0.84 ± 0.58	0.834 (−0.38–0.31)	Wilcoxon signed-rank

## Data Availability

The datasets used and/or analyzed during the current study are available from the corresponding author upon reasonable request.
